# EEG patterns of self-paced movement imaginations towards externally-cued and internally-selected targets

**DOI:** 10.1038/s41598-018-31673-2

**Published:** 2018-09-06

**Authors:** Joana Pereira, Andreea Ioana Sburlea, Gernot R. Müller-Putz

**Affiliations:** Institute of Neural Engineering, Graz University of Technology, Graz, Austria

## Abstract

In this study, we investigate the neurophysiological signature of the interacting processes which lead to a single reach-and-grasp movement imagination (MI). While performing this task, the human healthy participants could either define their movement targets according to an external cue, or through an internal selection process. After defining their target, they could start the MI whenever they wanted. We recorded high density electroencephalographic (EEG) activity and investigated two neural correlates: the event-related potentials (ERPs) associated with the target selection, which reflect the perceptual and cognitive processes prior to the MI, and the movement-related cortical potentials (MRCPs), associated with the planning of the self-paced MI. We found differences in frontal and parietal areas between the late ERP components related to the internally-driven selection and the externally-cued process. Furthermore, we could reliably estimate the MI onset of the self-paced task. Next, we extracted MRCP features around the MI onset to train classifiers of movement *vs*. rest directly on self-paced MI data. We attained performance significantly higher than chance level for both time-locked and asynchronous classification. These findings contribute to the development of more intuitive brain-computer interfaces in which movement targets are defined internally and the movements are self-paced.

## Introduction

Brain-computer interfaces (BCIs) provide a way of interaction with the external world by replacing the brain’s neuromuscular output pathways. This is particularly interesting for persons who suffer from severe motor disabilities^1,2^. Researchers have been working with BCIs based on electroencephalography (EEG) with the goal of helping individuals with motor impairments, like people with amyotrophic lateral sclerosis, spinal-cord injury (SCI) or stroke survivors^2^. Typical BCI control strategies involve sustained movement imagination (MI) of squeezing a ball, repetitive opening and closing of the hand, or repeated feet MI. These tasks typically induce a power decrease or increase of EEG activity in certain frequency bands^3^. BCIs which exploit the modulation of these rhythms of the sensorimotor cortex are known as SMR-based BCIs, and have been successfully used in asynchronous BCI scenarios^4–7^. Despite their success cases, the strategies used (e.g. repetitive feet MI) often do not correspond to the actual movements executed by the end-effector (e.g. hand close). Ideally, a BCI would accurately comprehend and mimic the way one plans a movement, allowing for a more natural control^8^. In this study, we use EEG to investigate the neurophysiological signature of the processes that lead to a single reach-and-grasp MI. In our view, this single motor task offers a more natural mental strategy which could be used by tetraplegic end-users to trigger a grasping command of a neuroprostheses^9^.

Reaching and grasping a glass among many others located on a table involves interacting neural processes which cover the domains of both perceptual and movement-related decision making. First, the surrounding environment needs to be perceived and sensory information processed. Allocating attention to the environment and defining one of the glasses as a target remains within the domain of perceptual decision making. These processes are not strictly motoric, since they define the target of movement, rather than the movement itself. Second, there must be preparation and planning for the actual movement. Namely, the movement onset and other movement details, like speed or shaping of the hand, are defined^10,11^.

Regarding the definition of the movement target, when more than one target is available, it is necessary to decide among various alternatives. If the target is defined by direct influence of an external stimulus, this process is externally-cued. Externally-cued processes are common on cue-based BCI training paradigms. However, in a BCI final application on daily-life scenarios, the target is often defined by an internally-driven process, without direct influence of an external cue. Therefore, it is important to determine whether these two processes are differently represented in the EEG (and if so, how?). The neural activity underlying perceptual and cognitive processes prior to motor tasks can be investigated using event-related potentials (ERPs). Recently, it has been shown that there is a modulation of ERPs’ later components, like the P300, as a function of complexity of cognitive control prior to motor tasks^12^. The P300 is a large positive component which peaks between 350–600 ms after stimulus onset^13^. For task-relevant stimuli, the P3b subcomponent is particularly relevant, since it is often related to the cognitive resources allocated to a certain goal-directed task^13,14^. There is evidence that the P300 peak is related with stimulus evaluation, but independent from the upcoming response^15^. While the P300 peak seems to be task-independent, the following negative-going phase has been related to decision-making prior to the preparation of a response^16,17^. Furthermore, this phase has been reported to be most conspicuous and durable with increased level of task complexity prior to a button press^12^.

For natural neuroprostheses or robotic arm control, the users should also have the possibility to initiate the movement at their own pace, the BCI being responsible to detect and/or classify that movement intention asynchronously. Movement-related cortical potentials (MRCPs) are known to reflect the cortical processes employed in movement planning and execution^18,19^ and can be used for movement detection and/or classification. MRCPs are time-domain amplitude modulations in the delta frequency band which occur around movement execution (ME), MI or attempted ME^18,19^. When associated with self-paced tasks, these modulations are known as Bereitschaftspotential (BP)^19^ and are characterized by a slow negative deflection which begins several hundreds of milliseconds before the movement onset. Its peak negativity is reached near the onset, followed by a return to baseline level^20^. Since MRCPs encode several movement properties (e.g. different grasp types, force levels or speed)^21–31^, and its detection has a short latency^32,33^, there is growing interest in using MRCPs for BCI control. Still, analyzing and exploiting the MRCPs around the onset of a self-paced MI is challenging. On a self-paced MI task, it is not possible to align the EEG activity to a cue, nor to a measurable myographic or kinematic onset. This alignment is important if one wants to extract MRCP features to train movement detectors (i.e. classification of movement *vs*. rest). For that reason, researchers have trained detectors on self-paced ME features, in which there is a measurable movement onset, to later detect self-paced MI^30,31,33^. This approach is not suitable for users who do not have residual muscular activity on their upper limbs. In that case, one would have to train the detectors directly on self-paced MI features.

In this study, we conducted an experiment in which healthy participants performed a self-paced single reach-and-grasp MI towards one of five targets displayed on a screen. The participants defined their target either by the direct influence of a cue, or by an internally-driven selection process. After defining their target, the participants could then freely decide when to start the reach-and-grasp MI. Similar to Libet’s experiments^34^, we estimated an MI onset which reflected the participants’ awareness of their intention to perform the reach-and-grasp. Our first hypothesis is that the EEG is modulated differently, depending on whether the target was externally-cued or internally-driven. We expect to find these differences in the late ERP components following target presentation, since they reflect cognitive control prior to the preparation of the motor task. Our second hypothesis is that it is possible to train detectors directly on self-paced MI features, when using this estimated MI onset. The performance of the detector, which was based on MRCP amplitude features, was evaluated offline in both time-locked and asynchronous scenarios. Understanding the neural processes of MI tasks, in which movement targets are defined internally and the movements are self-paced, can help us to develop more intuitive BCIs.

## Results

### Experimental paradigm

**Figure 1:**
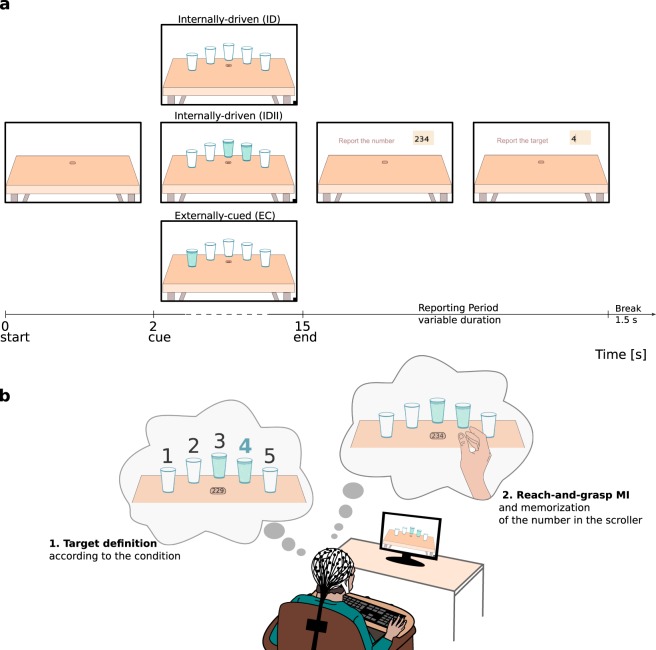
Experimental paradigm. (**a**) Trial timings and structure. A trial started with a 2 s baseline period. Thereafter, one of the cues associated with one of the three experimental conditions was displayed. In the internally-driven (ID) condition, all glasses were empty and subjects chose freely one of the five glasses. In the second internally-driven (IDII) condition, subjects chose one of the two glasses filled with water. In the externally-cued (EC) condition, the target was the one glass filled with water. (**b**) Experimental setup and task illustration. After defining the target according to the condition, subjects performed the MI of a reach-and-grasp directed towards the glass at their own pace. We positioned a scroller with numbers on the middle of the screen and instructed the subjects to memorize the number that was on the scroller when they perceived the urge to perform the MI. At the end of each trial, there was a reporting period in which subjects reported the number they memorized and the selected target. In this figure the example corresponds to the IDII condition.

We recorded the EEG and electrooculography (EOG) signals of 15 healthy human subjects while they were performing the paradigm illustrated in Fig. 1. Each session consisted of runs in which visual cues were displayed on a computer monitor. An average of 6.6 ± 0.7 (s.d.) runs were recorded per subject. A run was composed of trials in which the subjects were instructed to define the movement target according to the condition and then perform the MI of a single reach-and-grasp directed towards that target, in a self-paced manner (Fig. 1b).

In all conditions, after a baseline of 2 s, five glasses showed up in addition to a scroller that showed consecutive 3-digit numbers every 750 ms. The scroller was positioned at the center of the screen and the glasses could appear to have water or to be empty. On the internally-driven condition (ID) the participants could freely select one of the five glasses. On the second internally-driven condition (IDII) the participants could select one of the two glasses which had water. On the externally-cued condition (EC) they were asked to perform the task towards the only glass which had water. The glasses with water in both EC and IDII conditions were pseudo-randomly positioned and all glass positions were covered with the same frequency. We instructed the participants to define the target according to the condition and as soon as they saw the glasses. After defining the target, subjects could perform the kinesthetic MI of a single reach-and-grasp towards the glass at their own pace. To help the participants to perform the MI, they had the possibility to execute real reach-and-grasps towards a glass at the beginning of the experiment. Inspired by Libet’s experiments^34^, we instructed the subjects to memorize the number that was on the scroller when they perceived the urge to perform the MI. At the end of each trial, subjects reported the target (from 1 to 5) and the number that was displayed on the scroller. Figure 1a shows the trial timings and exemplifies the reporting on a IDII condition. A new trial started after a break of 1.5 s. An average of 195 ± 25 (s.d.) trials and 3 additional rest runs (with duration of 60 s each) were recorded per subject.

We analyzed the channel-space and source-space EEG during the target definition period by time-locking to the cue and grouping the trials in the 3 conditions. Henceforth, we refer to this section as the cue-locked EEG. Furthermore, we analyzed the EEG around the MI by time-locking to the onset which was estimated using the numbers reported by the participants. Henceforth, we refer to this section as the response-locked EEG. We used the MRCP features around the estimated onset to train and evaluate time-locked and asynchronous detectors of the self-paced MIs.

### Cue-locked EEG

**Figure 2:**
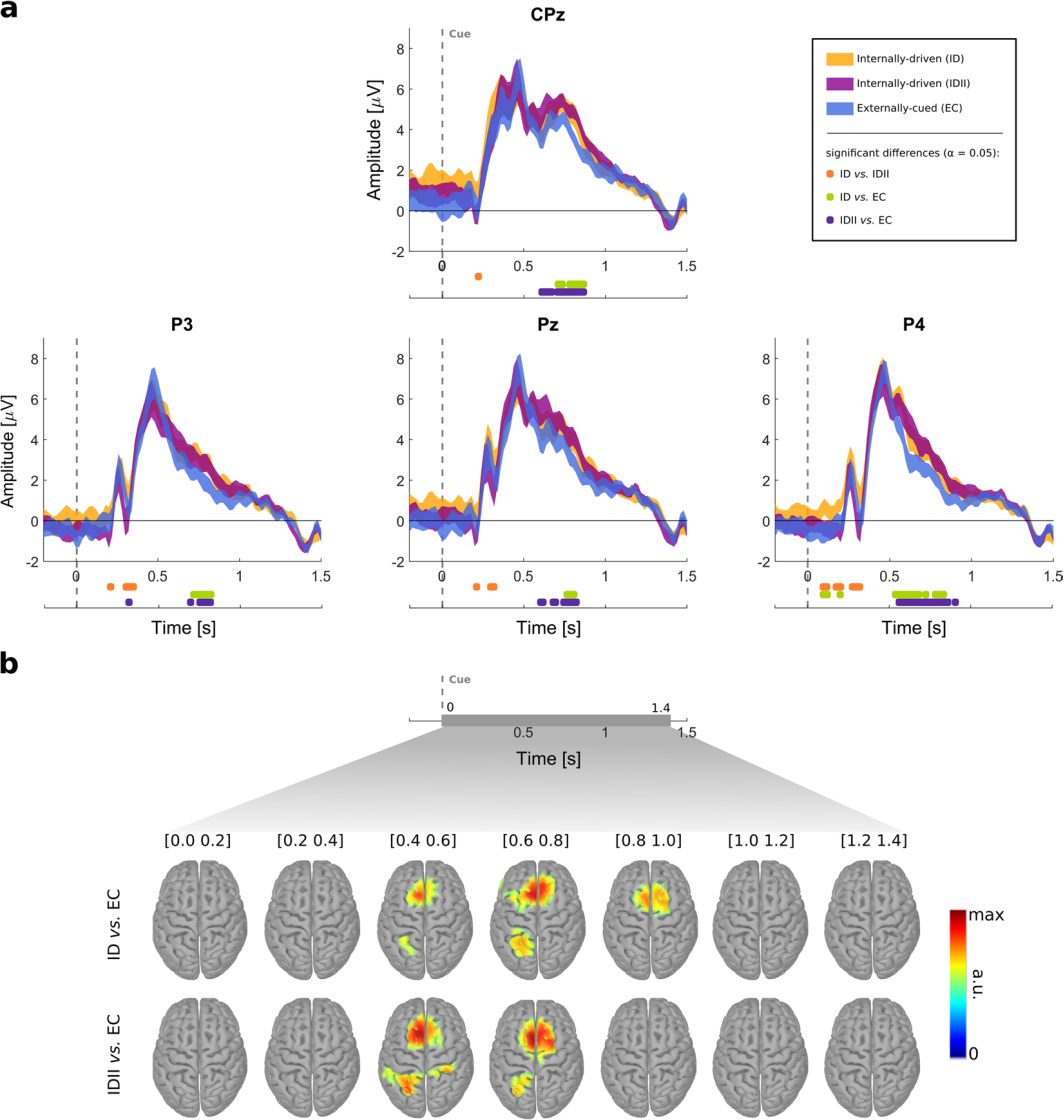
Cue-locked EEG. (**a**) Grand-average ERPs over electrodes CPz, P3, Pz and P4 (0 s corresponds to the cue). Colored shaded areas show the 95% confidence interval for the mean (*α* = *0*.*05*) of each experimental condition. Significant differences between the conditions are color-coded and presented below each channel plot (assessed with paired-sample two-tailed permutation tests based on *t*-statistics, *α* = *0*.*05*). (**b**) Differences between ERPs in the source space (sLORETA) from 0 to 1.4 s with respect to the cue. Sources were averaged on non-overlapping time-windows of 200 ms. We show the significantly different voxels between ID and EC conditions, and IDII and EC conditions (paired-sample two-tailed permutation tests based on *t*-statistics, *α* = *0*.*05*). No significant differences were observed on the source maps between ID and IDII conditions.

We analyzed the EEG on the target definition period by grouping the trials in conditions and time-locking them to the cue (Fig. 1a, second 2). At this time point, the glasses were displayed and the subjects defined their target according to the condition. Grand-averaged cue-locked ERPs for the conditions (ID, IDII and EC), as well as points in which statistically significant differences among the conditions were found, are shown in Fig. 2a for selected channels over centro-parietal areas. Supplementary Figure I additionally shows the grand-averaged ERPs over a larger set of channels. For all conditions, we found two distinctive peaks. We observed an earlier positive peak at ~200 ms which was more prominent in parietal and occipital electrodes. A second positive peak emerged at ~500 ms with higher amplitude over centro-parietal electrodes. The location and timing of this second peak are consistent with the P300 component, concretely with the P3b subcomponent. This peak was followed by a slow negative-going phase, reaching the baseline ~1.3 s after the cue on parietal electrodes. A third peak could be identified in more frontal and central areas which overlapped this negative-going phase of the P300.

We found significant differences (α = *0*.*05*, paired-sample two-tailed permutation tests based on *t*-statistics) in the earlier ERP components around 250 ms after cue, between the ID conditions (ID *vs*. IDII). Concretely, earlier ERP amplitudes were higher for the ID condition, when comparing with the IDII condition. On the negative-going phase that followed the P300 peak, we found significant differences (α = *0*.*05*, paired-sample two-tailed permutation tests based on *t*-statistics) between the EC and the ID conditions (ID *vs*. EV and IDII *vs*. EC). Namely, ERP amplitudes associated with the ID and IDII conditions were significantly higher from ~600 to 800 ms after the cue, when comparing to the EC condition. No significant differences were found on the late ERP components related to both ID conditions (ID *vs*. IDII).

We then investigated these differences in the source space. The results of the source analysis, in which the inverse problem was solved using standardized low-resolution brain electromagnetic tomography (sLORETA)^35^, are shown in Fig. 2b. Differences were investigated in a time-window of [0 1.4] s with respect to the cue. We averaged non-overlapping 200 ms segments for each condition and determined the significantly different voxels among conditions. On Fig. 2b only significant voxels are colored. Significant differences (α = *0*.*05*, paired-sample two-tailed permutation tests based on *t*-statistics) were present from 0.4 to 1 s between ID and EC conditions (ID *vs*. EC and IDII *vs*. EC). Namely, we observed increased activations on frontal-central and posterior parietal areas on the ID conditions. No significant differences were found between ID conditions (ID *vs*. IDII).

### Response-locked EEG: MRCPs

**Figure 3:**
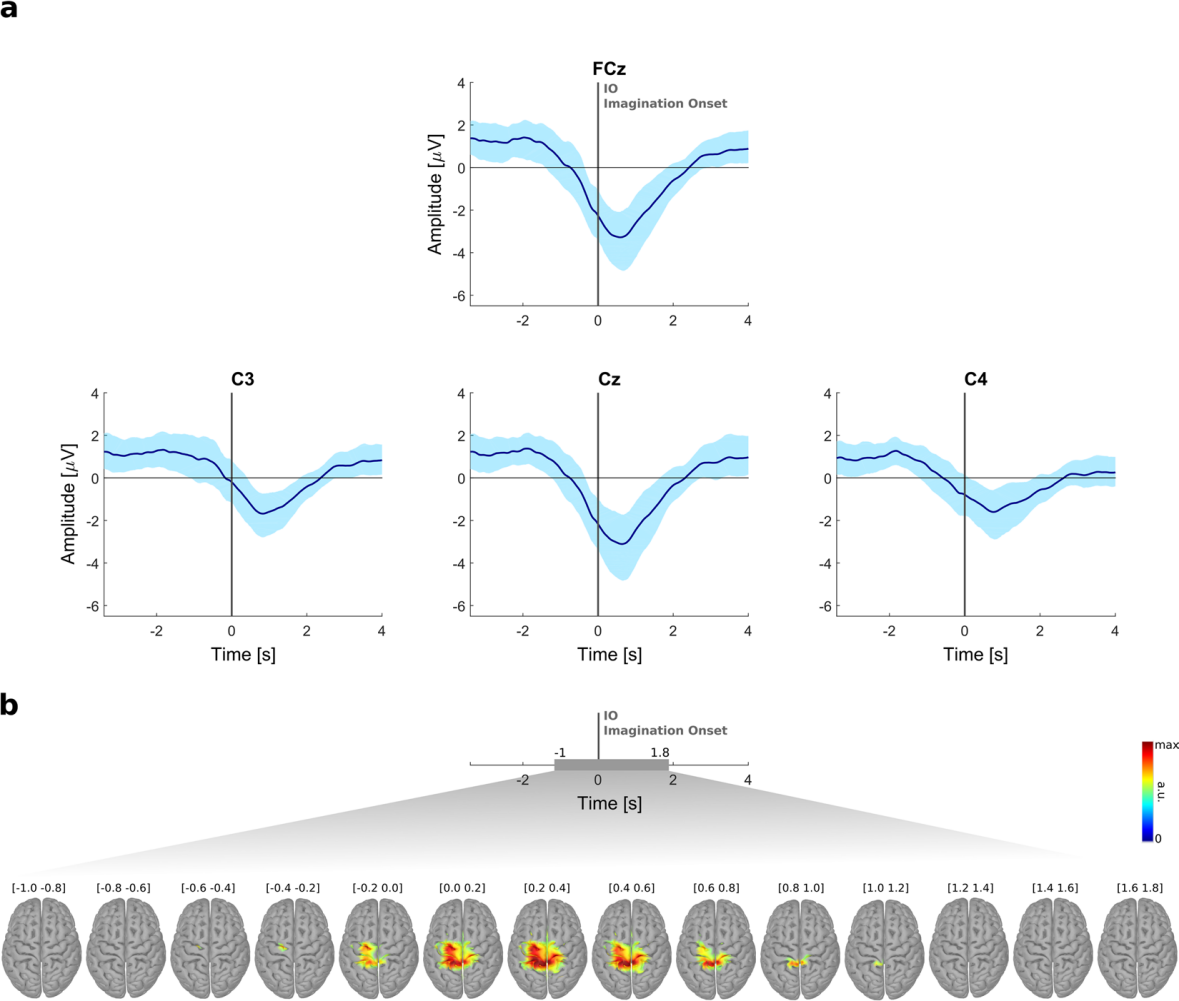
Response-locked EEG. (**a**) Grand-average MRCPs on electrodes FCz, C3, Cz, and C4 (0 s corresponds to the imagination onset, IO. In light blue we show the 95% confidence interval for the mean (*α* = *0*.*05*). (**b**) Grand-average MRCP on the source space from −1 to 1.8 s with respect to the IO. Source maps were averaged on non-overlapping time-windows of 200 ms and only significant voxels with respect to a baseline period are shown (assessed with paired-sample two-tailed permutation tests based on *t*-statistics, *α* = *0*.*05*).

After defining their movement target, the subjects performed a self-paced MI of a single reach-and-grasp. By time-locking to the number reported by the participants, we obtained an imagination onset (IO), which enabled us to analyze the low-frequency EEG modulations around the MI. We observed potentials in the delta frequency band consistent with MRCPs, concretely with the BP which is characteristic of self-paced motor tasks. MRCPs were observed in 14 out of the 15 subjects. The grand-average MRCPs are shown in Fig. 3a for selected channels over the motor cortex. Supplementary Figure II shows the MRCPs on a larger set of channels. We observed a negativity that started at around 1 s before the IO and culminated on a negative peak that was higher in absolute amplitude in the midline electrodes (FCz and Cz) at 500 ms after the IO. This peak negativity was followed by a slow return to baseline level, which happened ~2 s after the IO. From a single-subject perspective, there was variability in peak amplitude and respective latency, which can be seen in Supplementary Table I. Negative peak amplitudes ranged from −1 to −14 *μ*V and occurred from −0.4 to 1.4 s with respect to the IO. In Supplementary Table I, the amplitude and latency of the peak negativity for subject s10 are not reported, since for this subject we did not observe the characteristic negative slope, when time-locking to the IO.

We analyzed the MRCPs in the source space on a time-window of [−1 1.8] s with respect to the IO. Grand-averaged sLORETA source maps are shown in Fig. 3b, in which only significant voxels (α = *0*.*05*, paired-sample two-tailed permutation tests based on *t*-statistics) with respect to the baseline ([−2.2 −2] s) are colored. Compared to baseline, the estimated activity was first significant (0.4 s before IO) at the supplementary motor area (SMA) and premotor cortex, becoming broader on remaining motor areas (primary motor cortex and primary somatosensory cortex) and posterior parietal cortex, when closer to the peak negativity (0.2 to 1 s after IO).

### Time-locked classification

**Figure 4:**
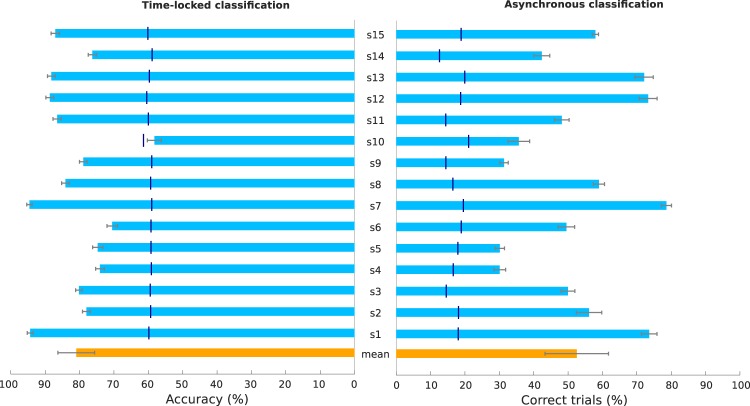
Classification results. Left: Time-locked classification accuracy. We show the mean of the 10 × 5-fold cross-validated accuracy and respective confidence interval (*α* = *0*.*05* per subject. The orange bar represents the grand-averaged accuracy. The horizontal dark blue lines (or orange lines, in the case of the mean) over each bar represent the chance level (*α* = *0*.*05*, adjusted Wald interval^57^). Right: Asynchronous classification performance. We show the mean of the 10 × 5-fold cross-validated percentage of correct trials and respective confidence interval (*α* = *0*.*05*) for each subject. The horizontal dark blue lines over each bar represent the chance level (obtained by permuting the labels 500 times). The orange bar represents the grand-averaged results.

We performed two types of offline single-trial classification to detect the MRCPs associated with the self-paced MI task: time-locked classification and, to simulate an online scenario, asynchronous classification. For both, a shrinkage regularized linear discriminant analysis^36^ classifier was used to discriminate MI *vs*. REST classes. The results of the 10 × 5-fold cross-validated time-locked classification are summarized in Fig. 4, on the left bar plot. This plot shows the mean accuracy per subject and respective confidence intervals. With the exception of subject s10, all subjects were above chance level. Notably, 8 of the 15 subjects had accuracies above 80%. Overall, a classification accuracy of 81.2 ± 9.5% (mean ± s.d.) was achieved. The average true positive rate (TPR) was 81.0 ± 9.7% and the average false positive rate (FPR) was 19.1 ± 9.7%. Supplementary Table I additionally shows the TPR, FPR, false negative rate (FNR) and true negative rate (TNR) per subject.

### Asynchronous classification

The results of the 10 × 5-fold cross-validated asynchronous classification are summarized in Fig. 4, on the right bar plot. We performed a trial-based evaluation over a 12 s long window which started 1 s after cue presentation. We labeled the MI class as the positive class (3 of the 12 s of the trial were labeled as MI), and the REST class (remaining 9 s) as the negative class. The performance measure was the number of correctly classified trials. A trial was only considered correct when there was at least one MI detection within the MI period (i.e. at least one true positive) and no detections during REST (i.e. no false positives). Figure 4 (right bar plot) shows the percentage of correct trials per subject and its respective 95% confidence interval. Chance-levels were determined by structured permutation of the labels. The average percentage of correctly classified trials is at 52.5 ± 16.8%, and all subjects had performances above chance level. Supplementary Table I additionally shows the positive likelihood ratio (PLR) per subject, which corresponds to the ratio between the TPR and FPR.

**Figure 5:**
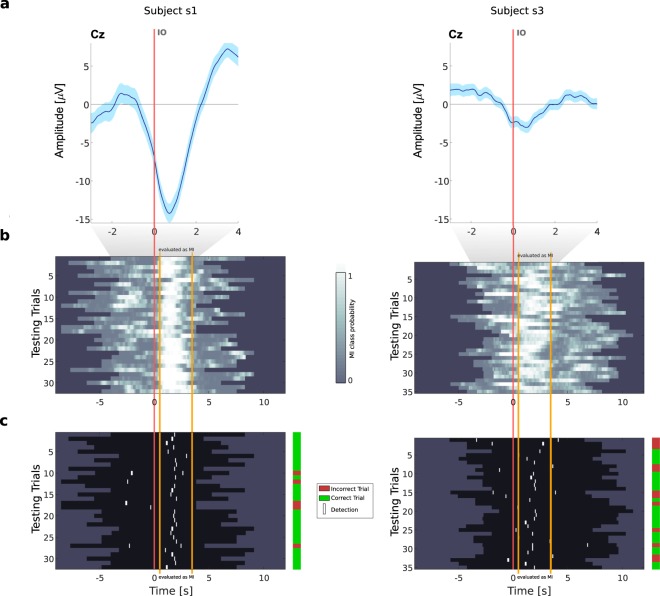
Trial-based asynchronous evaluation examples. We give two examples of classifier outputs and respective movement detections on a testing fold of a subject with performance above average, subject s1 (left panel), and for representative subject s3 (right panel). (**a**) MRCPs on channel Cz. (**b**) Single-trial image showing the normalized probability for the MI class over time (time-locked to the IO). (**c**) Single-trial image showing the corresponding detections according to the evaluation criteria applied. Movement detections are marked in white over each trial (trials are represented in black). The orange vertical lines mark the beginning and the end of the period evaluated as MI, second 0 is the IO. The vertical bar positioned right to each of the single-trial images shows the trials marked as correct (green) and incorrect (red). A trial was only considered correct when there was at least one true positive (detection within the MI period, limits marked in orange) and no false positives (no detections outside MI period).

In Fig. 5 we give two examples of testing folds for subjects s1 and s3, to better illustrate the trial-based evaluation procedure of the asynchronous classification. Figure 5a shows the MRCPs on channel Cz for those two subjects. We then show the single-trial classifier probabilities for the MI class over time on a testing fold (Fig. 5b). Figure 5c represents the effect of our evaluation criteria for the performance on that fold, in terms of correct and incorrect trials. Subject s1 had a performance above average (74% correct trials). This performance was driven by the consistently higher classification probabilities of the MI class during the MI period, when compared to the REST period (Fig. 5b). We attained very few false positives and a high number of true positives, when compared for instance to subject s3 (Fig. 5c).

### Behavioral analysis

**Figure 6:**
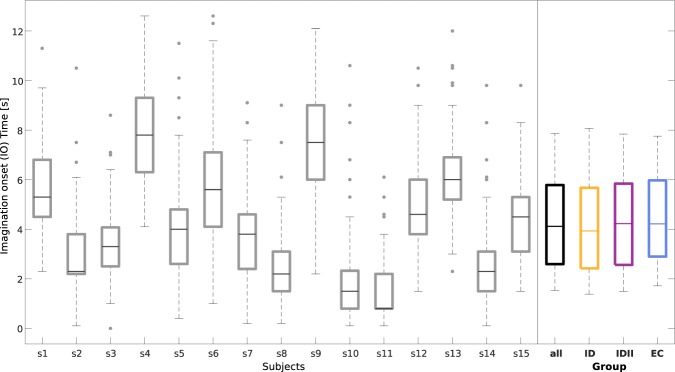
Imagination onsets relative to the cue. We show the boxplots depicting the time of the IOs per subject (in grey) and the boxplots with respect to the group results. For the later, we grouped the IOs in the three experimental conditions (in orange for the ID condition, in purple for the IDII condition, and in blue for the EC condition).

Since the subjects were performing the MI at their own pace, it is of interest to analyze the time of the IOs for each subject. Figure 6 shows the boxplots graphically depicting the time of the IOs per subject. As expected from the paradigm and instructions given to the participants, there was variability in IOs within and among the subjects. This figure also shows the group boxplots correspondent to the group-average IO for the three conditions. To check for possible confounds, we analyzed whether there were statistically significant differences among the conditions. No statistically significant differences were found among conditions (α = *0*.*05*, paired Wilcoxon rank-sum test, FDR corrected for multiple comparisons).

## Discussion

We investigated the EEG correlates of a single reach-and-grasp MI task, in which movement targets were defined internally or by the influence of an external cue, and the onset of movement was self-paced. We found that the ERPs associated with the externally-cued process were significantly different from those of the internally-driven selection. The differences on the late ERP components were predominantly encoded in frontal and posterior parietal cortices. By time-locking to an estimated onset, we observed an EEG potential consistent in morphology and topographical distribution with an MRCP. MRCP features were then used to detect self-paced movement imaginations. The performance of the classifier, in both time-locked and asynchronous scenarios, shows that it is possible to detect MRCPs in single-trial when training solely on self-paced MI.

### Cue-locked EEG

The participants started each trial by defining the target of their reach-and-grasp MI. This target definition could have been externally-cued, in that the target was directly influenced by the cue, or internally-driven. We analyzed the cue-locked EEG following target presentation, to find whether the ERP components differed depending on whether the participants were, or not, externally-cued in respect to their target of movement. Our findings suggest that the P300 (specifically the P3b subcomponent) associated with the ID conditions is different from the EC condition. While the peak latencies and respective amplitudes were similar, significant differences were found from 600 to 800 ms after cue presentation. Concretely, the amplitudes of the negative slope following the P300 peak were higher for the ID conditions. The P300 is related to stimulus processing^15^, which was necessary in all experimental conditions. However, the P3b has also been previously related to cognitive resources allocated to a certain goal-directed task^13,14^. We observed that aside from the slow negative-going phase after the P300 peak, which lasted until 1.3 s after target presentation, there was an overlapping positive waveform on more central and frontal electrodes. These late positive components which overlap the negative slope of the P300 have been grouped under the label of positive slow waves^37^. While there are many interpretations about which processes are being encoded in these positive slow waves, Niewhenhuis and colleagues suggested that these components encode processes which guide the future response, in the light of what are the task demands and rules^16^. Moreover, this negative-going phase following the P300 peak has been related to decision-making prior to the preparation of a response^17^. These explanations are in line with our observations. In our task, the target could only be truly selected in the two ID conditions. One possible explanation is that the need of internally selecting a target evokes an increase in task demand which is reflected in higher amplitudes in the late ERP components. Since these late positive components are not strictly related to motor responses^37^, one topic of interest for future studies would be whether the differences observed in the ERP components are also present in similar tasks which do not involve motor planning.

It is important to note that subjects decided among glasses with water (IDII and EC condition) or empty glasses (ID condition). This constitutes a limitation of our study, concerning the paradigm design. However, the differences observed on the late ERP components cannot be explained by this empty *vs*. full context. Concretely, differences on the late ERP components were present on the ID *vs*. EC and IDII *vs*. EC condition. No differences were observed in the later components between the ID condition (in which participants freely selected an empty glass) and IDII or EC conditions (in which participants freely selected a glass with water).

Using source imaging, we estimated that the differences among the ID and the EC conditions were located in frontal and posterior parietal areas. The neural generators of the P300 are not clearly characterized, but our results are in line with previous studies that point out those areas as generators of the P300 component^38,39^. One explanation is that tasks which involve response to visual stimuli activate frontal areas that then transmit information to temporal and parietal areas. These areas are in turn responsible for indexing task context updates, which is a necessary step for response organization and production^16^. In our study, the information relates with the target of the upcoming motor response: the self-paced MI task.

### Response-locked EEG: MRCPs

After definition of the movement target, the participants performed a self-paced MI of a single reach-and-grasp. We introduced a scroller with numbers and asked the subjects to report the number that was displayed when they felt the urge to start the MI. With this strategy we estimated an MI onset, which enabled us to analyze the response-locked EEG activity associated with a self-paced MI task. We found a cortical potential consistent in timing, morphology, and topographical distribution with an MRCP. We observed a negative potential which started around 1 s prior to the IO and was more prominent in the central-medial scalp. The peak negativity of the BP is typically occurring closer to movement execution onset (when the onset is estimated using EMG). However, we observed that for the majority of the subjects, the peak negativity occurred a few milliseconds after the IO. The most obvious reason for such difference in latencies is that our alignment is not as accurate as an eventual ground truth: a new number was introduced on the scroller every 750 ms which is a significant time interval and can result in imperfect alignments. Moreover, it is important to notice that the start of the BP is known to vary among subjects^19^. Using source imaging, we were able to estimate which brain areas were active around the self-paced MI. It is important to point out that, despite recording the individual electrode positions, our head models were generated from a template magnetic resonance imaging scan, which can increase the location error of the estimated EEG sources. Our results indicate the involvement of SMA, premotor areas, M1, somatosensory cortex and posterior parietal cortex. All these areas are well known to be involved in movement planning and preparation. The SMA and premotor cortex have been indicated as the origin of the BP. For self-paced finger movements, it has been reported that SMA activation preceded that of the motor cortex by 800 ms^40^.

For the alignment of the trials to an MI onset, we used a memorization task instead of a motor execution task (such as a key press at the end of the MI) to avoid additional movement-related patterns which would interfere with the MI pattern, creating confounds. Furthermore, this intermediate memorization task allowed a separation between the target definition period and the actual MI. Recently, Aliakbaryhosseinabadi and colleagues^41^ showed that the MRCP features on a ME task changed significantly between single and dual-task (involving counting of auditory stimuli). The higher attention diversion imposed by the dual-task lead to a significant reduction in specific MRCP features, further affecting the performance of the movement intention detector. In our paradigm, there was a dual-task of MI and memorization. A decrease in MRCP features (e.g. decrease in absolute amplitudes of the peak negativity) is therefore likely and this can be seen as a drawback in our strategy. It is also relevant to mention that the morphology of MRCPs can be influenced by impairments of the sensory-motor system. As an example, on a cue-based protocol, absolute amplitudes of the peak negativity are reduced in a group of spinal cord injured individuals when compared to a group of healthy subjects^42^. Another study has shown that there is a larger variability of the MRCP in individuals with spinal cord injury (both within and between subjects)^43^. Furthermore, on self-paced ME, longer BP latencies have been observed in aged subjects (>60 years-old)^44^. For these reasons, it is necessary to investigate the suitability of our paradigm in end-users.

### Self-paced MI detection

We performed offline detection of self-paced MI using MRCP features. Detection based on MRCPs can be an alternative to SMR-based BCIs, which often involve less intuitive control strategies. MRCPs are modulated by several movement factors that, when correctly classified, could allow for new control strategies. These factors include movement speed^22,24^, force^24^, movements involving different joints^21,28^, grasp types^29^ and the presence of motor goals^27^. Additionally, the detection of MRCPs is faster, providing timely feedback to the user. This last fact could have implications for BCIs which aim to induce cortical plasticity^32^. However, the detection of MRCPs presents its challenges. A correct alignment to the movement onset is critical to train detectors based on MRCP features. Hence, researchers have trained detectors using MRCP features from self-paced ME, and later tested these detectors on self-paced MI^30,31,33^. This approach is only suitable for users who have residual muscular activity. In the current study, time-locking to the MI onset allowed us to train classifiers directly on MRCP features of self-paced MI data. This can be of particular interest for end-users without residual motor function of the upper limb.

Regarding the time-locked classification, an average accuracy of 81 ± 10% was achieved. With the exception of one subject, all remaining subjects were above chance level. While it is of interest to assess the performance of the time-locked classification, especially when testing new paradigms, the asynchronous classification is of greater interest since it corresponds more closely to the BCI scenario that we envision. Therefore, we performed asynchronous classification with a trial-based evaluation scheme and assessed the classifier performance using the percentage of correctly classified trials. We used a strict evaluation in which a trial was only considered correct if there was at least one true detection and no false detections. As expected, those subjects who achieved higher performances on the time-locked scenario were also achieving higher performances on the asynchronous scenario. On average, 53 ± 17% trials were correctly classified. One could prematurely consider this performance to be poor. However, due to the low ratio between positive and negative examples, the chance level is 20%, and all of the subjects had performances above chance level. It will be critical in future online implementations to tackle the possibilities for decreasing the number of false detections (an average of 40% of trials had false detections). We believe that there are two main reasons for such spurious detections. Firstly, in some of the trials, there could have been errors in the reporting of the onset, which lead to incorrect alignments. Secondly, spurious detections can be caused by artefacts, and in future experiments it is important to deal with artefacts online. One possibility would be to detect artefactual segments online and ignore the classifier output on these segments, or to correct the artefacts without the need of ignoring the classifier’s output.

A direct comparison between the performance of our self-paced MI detector and other movement detectors is difficult, due to the variability among tasks (upper/lower limb, self-paced/cue-based, ME/MI/attempted movements), classification scenarios (offline, online, simulated classification procedures), type of participants (healthy, stroke, spinal cord injury), and among the metrics used to assess performance (e.g. accuracy, number of correctly classified trials, true positive rate). Sburlea *et al*.^26^ obtained an average of 61% correctly classified trials when using MRCP features for continuous detection of pre-movement state in self-initiated walking from healthy subjects. In this study, EMG was used to align the trials to the movement onset and only pre-movement EEG features (i.e. before EMG onset) were used to train the detector. In another study, Jochumsen *et al*.^25^ showed that on average ~75% of grasp movements were correctly detected for ME and MI (in healthy) and attempted ME (in stroke patients) in a cue-based paradigm.

### Main findings and conclusion

EEG signals during a self-paced MI of a single reach-and-grasp were recorded and analyzed offline and in healthy participants. We analyzed the ERPs following target presentation, to investigate the underlying perceptual and cognitive processes prior to the MI. Concretely, we were interested in the differences between a internally-driven target selection and a externally-cued process, in which the target is defined by the direct influence of an external cue. After target definition, and around the estimated onset of the self-paced MI, we analyzed the MRCPs as correlates of movement planning. Our results show that the late ERP components associated to the internally-driven process are different from those of an externally-driven process. Moreover, we were able to exploit the MRCP features around the MI onset to train detectors of MI directly on self-paced data. These findings help us understand and exploit the EEG representations of a MI task, in which movement targets are defined internally and the movements are self-paced. Finally, the goal is to provide end-users with a BCI control strategy which feels natural and is suitable for neuroprostheses or robotic arm control.

## Materials and Methods

### Participants

Fifteen healthy participants (23 ± 2 (mean ± s.d.) years old, 8 males) took part in this study. Participants gave their informed consent and the study was conducted in accordance with the protocol approved by the ethics committee of the Medical University of Graz (approval number: 29-058 ex 16/17). All subjects were right-handed.

### Instructions

Subjects sat on a comfortable chair, in a shielded room, facing the computer monitor that was placed at a distance of 130 cm. Their arms were supported in both armrests. Additionally, a wireless keyboard was placed in front of them, at the same level as their elbows (Fig. 1b). They used the keyboard to report the numbers, after the end of each trial - as described in Fig. 1a. During the experiment, subjects were asked to keep their gaze in the center of the monitor and to avoid moving their eyes towards the selected target during the trial. We also instructed them not to execute any arm/ hand movements within the trial, and explained the difference between movement imagination and execution. The subjects were asked to minimize blinks and muscular artefacts when not on the reporting period or break. The negative effects of these artefacts on the EEG recordings were explained. Lastly, we observed the participants performing the task. During the rest runs (each 60 s long), the subjects remained at rest and fixated their gaze on the center of the monitor, which displayed the table and the scroller. Rest runs were recorded as follows: one run was recorded at the beginning, one in the middle, and other in the end of the measurement.

### Signal recordings

**Figure 7:**
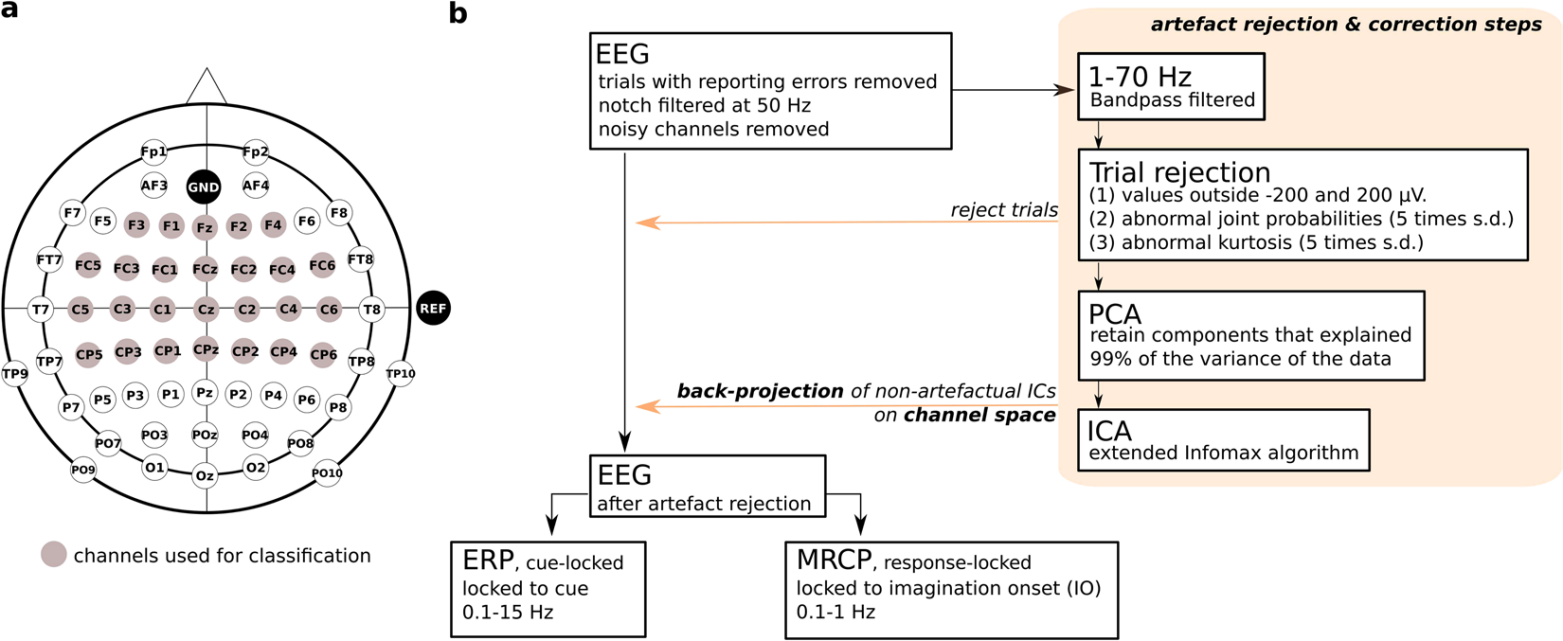
EEG recordings and processing. (**a**) EEG was recorded from 61 channels covering frontal, central, parietal, temporal and occipital areas. Highlighted are the channels which were later used for MI detection (classification of MI *vs*. REST). (**b**) Scheme representing the EEG processing steps.

EEG and EOG signals were recorded using 64 active actiCAP electrodes (BrainProducts GmbH, Germany). Reference was placed on the right mastoid and ground on AFz. The EEG was measured from 61 equally-spaced channels covering frontal, central, parietal, temporal and occipital areas (channel layout can be seen in Fig. 7a). Three EOG electrodes were placed above the nasion and below the outer canthi of the eyes. Additionally, we recorded the position of each EEG electrode in 3D coordinates using ELPOS ultrasound-based system (Zebris Medical GmbH, Isny im Allgäu, Germany). Biosignals were sampled at 1 kHz using two 32-channel BrainAmp amplifiers, and inspected using the BrainVision software (BrainProducts GmbH, Germany). For the recordings and time-synchronization we used the lab streaming layer framework (Swartz Center for Computational Neuroscience, UCSD, freely available online^45^). We used a photodiode to adjust the timestamps of the events to the moment when they were displayed on the monitor.

### Behavioral analysis

We discarded trials with incorrect responses from the rest of the analysis. In these trials, participants reported an incorrect number (i.e. which was not presented on the scroller during that trial) and/or incorrect target according to the condition (i.e. target which was not indicated by the EC or IDII conditions). An average of 9.4 ± 8.8 (mean ± s.d.) trials per subject had incorrect responses.

We used the reported numbers to determine the IOs. For that, we simply took the time point when the number was shown on the scroller. We assessed whether there were significant differences of IO times among the three conditions (ID, IDII and ED) using non-parametric Wilcoxon rank-sum tests, FDR corrected for multiple comparisons.

### Signal processing

EEG and EOG were processed offline using MATLAB 2015b (The MathWorks, Massachusetts, USA). We additionally used the BioSig toolbox^46^, EEGLAB^47^ and Brainstorm^48^. Figure 7b summarizes the EEG processing steps described in this section. After removing the trials due to incorrect responses, we removed noisy channels by visual inspection (3 EEG channels were removed on average). The data were notch-filtered (notch frequency of 50 Hz) to remove power line noise.

To deal with artefacts, we performed a trial-based artefact rejection followed by artefact correction using independent component analysis (ICA). For the trial-based artefact rejection, data was first band-pass filtered from 1 to 70 Hz with a zero-phase 4th order Butterworth filter. We then epoched the EEG with respect to the start of the trial and we used EEGLAB to: (1) find values outside an interval between −200 *μV* and 200 *μV*, (2) reject trials with abnormal joint probability and/or (3) abnormal kurtosis. A threshold of 5 times the standard deviation was used for each statistic. An average of 11.2 ± 4.2 (mean ± s.d.) trials were removed due to artefacts. In total, an average of 174.1 ± 24.8 trials remained for analysis (58 trials per condition). We then applied principal component analysis (PCA) for dimensionality reduction and retained components which explained 99% of the variance of the data. We used ICA on the PCA-compressed EEG and EOG data using the extended Infomax algorithm^49^. We marked the independent components that corresponded to ocular or muscular artefacts. Using visual inspection, an average of 11.6 ± 3.0 components per subject were marked as representing artefacts.

On the EEG data only subjected to notch filtering (i.e. no band-pass filter), the aforementioned artefact-contaminated trials were rejected, and the weights of the ICA decomposition were used to back-project exclusively the components without artefacts into the channel-space. For the three rest runs recorded, the ICA weights were also used for back-projection. We refer to the EEG in the channel-space after this artefact rejection and correction procedure as *cleaned EEG*.

### Cue-locked EEG

We analyzed the cue-locked EEG activity in the channel-space by filtering the cleaned EEG signals between 0.1 to 15 Hz with a 4th order zero-phase Butterworth filter. This band-pass filter offers a good compromise between removing noise and preserving information for P300 applications^50^. We then time-locked the trials to the cue, grouped them in conditions, and calculated the average. For each condition we calculated the confidence interval of the mean (α = *0*.*05*) using nonparametric *t*-percentile bootstrap statistics.

Differences among the conditions were assessed using nonparametric paired-sample two-tailed permutation tests based on *t*-statistics (α = *0*.*05*) on the [0 2] s window with respect to the cue^51^. This test was chosen since we performed individual tests at each time point and channel, and permutations tests can be used to mitigate the multiple comparisons problem^52^. For each permutation, paired samples *t*-statistics were obtained and the multivariate *t*-statistics *tmax* was calculated (*tmax* denotes the most extreme positive or negative value of all the *t-*scores across the entire family of tests). After all permutations, a *tmax* reference distribution was obtained. The *p*-values of each comparison were derived from this reference distribution, which is automatically adjusted to reflect the chance of false discoveries^53,54^. Since we conducted two-tailed tests at α = *0*.*05*, we found the critical values in the *tmax* reference distribution that cut off 0.025 on each tail. Each of the individual *t-*statistics was compared with those critical values to make a determination of significance: points falling above or below these critical values were declared statistically significant.

The differences among conditions were further investigated in the source space. For this purpose, we computed boundary element head models with OpenMEEG^55^ using the ICBM152 brain model template included in Brainstorm^48^ and the subject individual EEG electrode positions. The registration between the ICBM152 template (head model based on a non-linear average of 152 subjects) and the individual EEG electrode positions was done using the Brainstorm^48^ automatic alignment algorithm. This alignment is based on the three fiducial points (nasion, left ear and right ear) and a further iterative algorithm which finds a better fit between the two head shapes, using the remaining head points to improve the initial registration. We visually inspected this alignment. The cleaned EEG was downsampled to 250 Hz and band-pass filtered from 0.1 to 15 Hz. Three rest runs were also equally processed, and used to obtain full noise-covariance matrices with shrinkage regularization^56^. Finally, to solve the inverse problem, we computed 15002 brain sources using sLORETA^35^ with unconstrained dipole orientations. This means that sources were estimated independently for the three dipoles with orthogonal directions. To show the activity maps, the norm of the vectorial sum of the three orientations at each vertex was taken. Since we wanted to estimate the brain areas associated with the differences observed after the cue, we selected a time-window of interest, from [0 1.4] s. We then averaged the difference over conditions on non-overlapping segments with 200 ms for each subject and determined the significantly different voxels (i.e. the norm of the vectorial sum) using paired-sample two-tailed permutation tests based on *t*-statistics (α = *0*.*05*).

### Response-locked EEG: MRCPs

MRCPs were calculated by filtering the cleaned EEG signals between 0.1 to 1 Hz with a 4th order zero-phase Butterworth filter. We then time-locked the trials to the IO and calculated the subject averages. Grand-averages over subjects were obtained, as well as the respective confidence interval of the mean (α = *0*.*05*) using nonparametric *t*-percentile bootstrap statistics. Additionally, we analyzed the low-frequency EEG signals in the source space using the same methods for calculating the head models and solving the inverse problem as described in the section above. The cleaned EEG signals were downsampled to 250 Hz and bandpass filtered between 0.1 and 1 Hz. Three rest runs were also equally processed, and used to obtain full noise-covariance matrices with shrinkage regularization^56^. A time-window between [−1 1.8] s with respect to the IO was selected and the subject individual sources were averaged on non-overlapping segments with 200 ms. We computed the significantly different voxels with respect to a baseline [−2.2 −2] s using nonparametric paired-sample two-tailed permutation tests based on *t*-statistics (α = *0*.*05*).

### Time-locked classification

**Figure 8:**
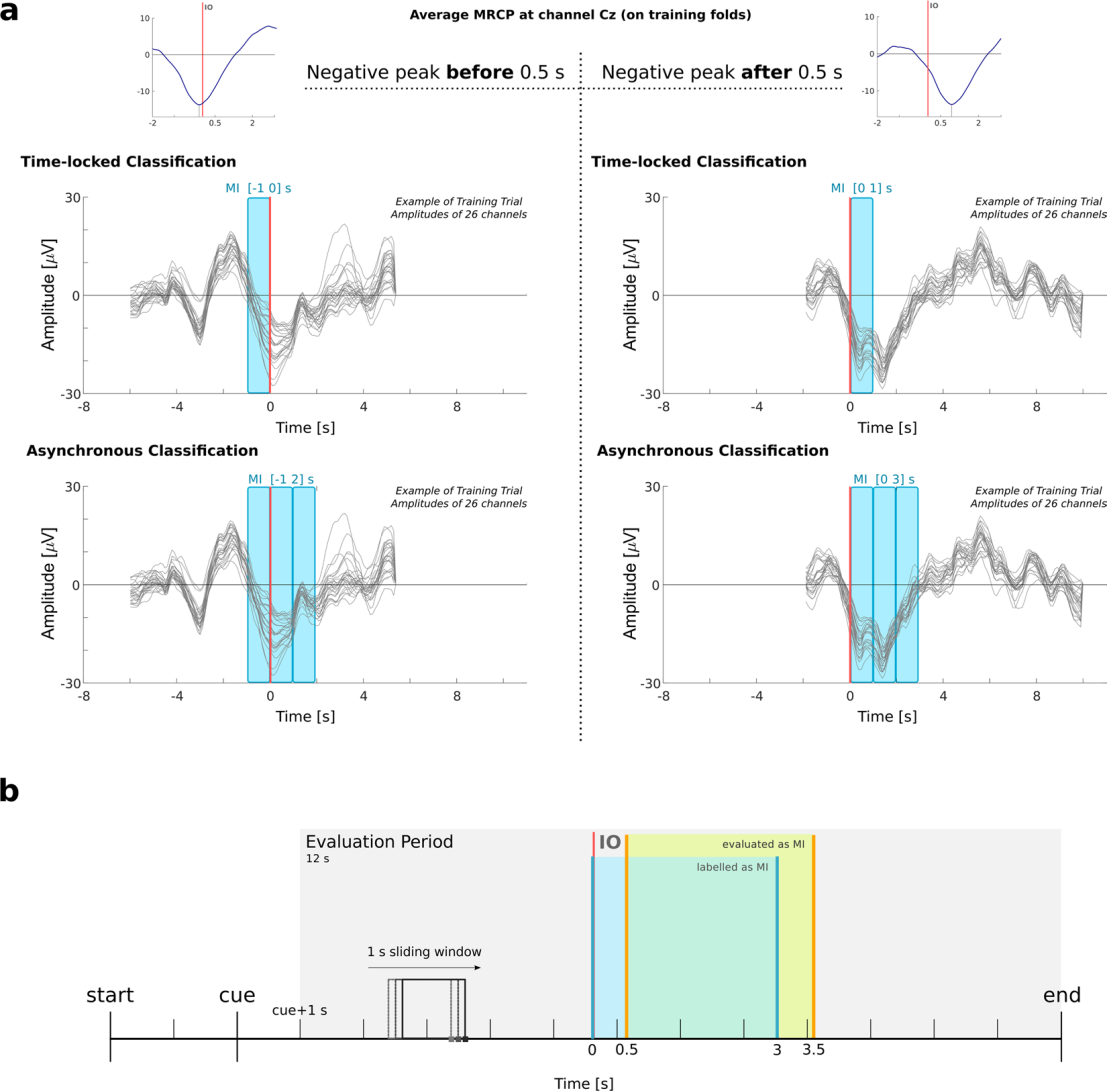
Illustration of the data segments used to train and to test the classifiers. (**a**) Scheme representing the definition of the MI window for feature extraction. We calculated the average MRCP at channel Cz on the training folds. We then searched for the latency of the peak negativity. For the time-locked classification, if the latency was smaller than 0.5 s, the MI window was [−1 0] s, otherwise [0 1] s was taken. For the asynchronous classification, if the peak was before 0.5 s, then three windows were taken from [−1 0], [0 1] and [1 2], otherwise [0 1], [1 2] and [2 3]. (**b**) Asynchronous classification and trial-based evaluation. On the 12 s of the evaluation period, we applied a 1 s sliding window on every sample. In blue we highlight an example of the period labeled as MI, and in orange the 0.5 s difference caused by the window definition.

MRCP features were extracted for movement detection (classification of MI *vs*. REST). In this section, we describe the pairwise comparison which was performed in the time-locked trials (i.e. in a synchronous manner). To extract the relevant time-domain amplitude features, we applied a zero-phase anti-aliasing filter and downsampled the data to 10 Hz to reduce computational effort. Then, data were processed as described in the previous section (0.1–1 Hz band-pass filtered cleaned EEG). We took the amplitudes over 1 s windows of 26 channels located over the somatosensory and motor areas relevant for the task (channels used are specified on Fig. 7a). For each subject, the set of EEG features was divided into training and testing on a 10 × 5-fold cross-validation procedure. For each trial, REST and MI features were extracted from time-windows with duration of 1 s, as follows:

- REST window. The start of the REST window varied among trials. As long as the window upper limit would not exceed the end of the trial, its start was at 4 s after the IO. Otherwise, the REST window started at −5 s.
- MI window. In order to determine the MI window, we had to take into account the differences in the MRCPs latencies among subjects. Therefore, we calculated the average MRCP on channel Cz on the training set data and searched for its peak negativity around [−2 2] s with respect to the IO. If the peak latency was before 0.5 s, we chose to start the MI window at −1 s. Otherwise, we chose to start the MI window at 0 s. The definition of the MI window is illustrated in Fig. 8a.

We classified the EEG with a shrinkage regularized linear discriminant analysis^36^, which is a state-of-the-art method for classification of event-related potentials^36^, and has been used in movement detection and classification of MRCPs^27–29^. The accuracies reported correspond to the average over all cross-validation procedure. Subject-specific chance-level was calculated based on the number of trials using the adjusted Wald interval^57^.

### Asynchronous classification

For the asynchronous classification, we took the amplitudes over the same 26 channels as with the time-locked classification, but used more training observations since we extracted three consecutive windows of 1 s each, per trial and per class. The criteria to define the start of the first window were the same as for the time-locked classification. This means that we chose for the REST class three (1-s long) windows between [4 7] s or [−5 −2] s, depending on the trials. For the MI class we chose three windows between [−1 2] s or [0 3] s, depending on the subjects.

The performance of the classifier was obtained in a 10 × 5-fold cross validation procedure, in which, for each repetition, 3 folds were used for training, 1 fold was used as validation, and 1 fold was used for testing. We performed a trial-based evaluation which was done on the trials of the testing set, starting 1 s after cue presentation. This led to an evaluation period of 12 s per trial, which is represented in Fig. 8b. The classifier was continuously evaluated with a sliding window of 1 s length, on every sample. For each sample *s*_*i*_, we took features from [*s*_*i*_ − 1, *s*_*i*_]. If at least half of the window (0.5 s) was within the MI period (indicated in blue in Fig. 8b), and the MI class probability was higher than 0.5, then this would be considered as a true positive. This explains the [MI_start_ + 0.5, MI_end_ + 0.5] s period highlighted in orange in Figs 5, 8b and Supplementary Fig. III.

The validation set was used to optimize a parameter: the number of consecutive detections (*x*). An MI was detected in sample *s*_*i*_ if that sample and all the previous *x* consecutive samples (i.e. [*s*_*i−x*_ *s*_*i*_]) would have been classified as positive. We varied *x* between 5 and 15, and chose the number which maximized the number of correctly classified trials on the validation set. A trial was correct when MI events were detected within the MI period (i.e. at least one true positive), and no MI events were detected during the rest (i.e. no false positives). Any other possibility was considered as incorrect. The chance level was calculated by permuting the IO 500 times (which lead to a structured permutation of the labels) and by repeating the described cross-validation procedure.

## Electronic supplementary material

Supplementary Information

## Data Availability

The datasets generated during the current study are available from the corresponding author on reasonable request.
