# Supplementary material for: EEG patterns of self-paced movement imaginations towards externally-cued and internally-selected targets
Source: Sci Rep. 2018 Sep 6;8:13394. doi: 10.1038/s41598-018-31673-2 (PMC6127278; doi:10.1038/s41598-018-31673-2)
Supplement: Supplementary file 1 — Supplementary Information [file 41598_2018_31673_MOESM1_ESM.pdf]

Supplementary Materials

EEG patterns of self-paced movement imaginations towards externally-cued and internally-selected targets

Joana Pereira [1], Andreea Ioana Sburlea [1], Gernot R. Müller-Putz [1*]

[1] Institute of Neural Engineering, Graz University of Technology
Graz, Austria
*Correspondence should be addressed to GMP (e-mail: gernot.mueller@tugraz.at)

Supplementary Figures

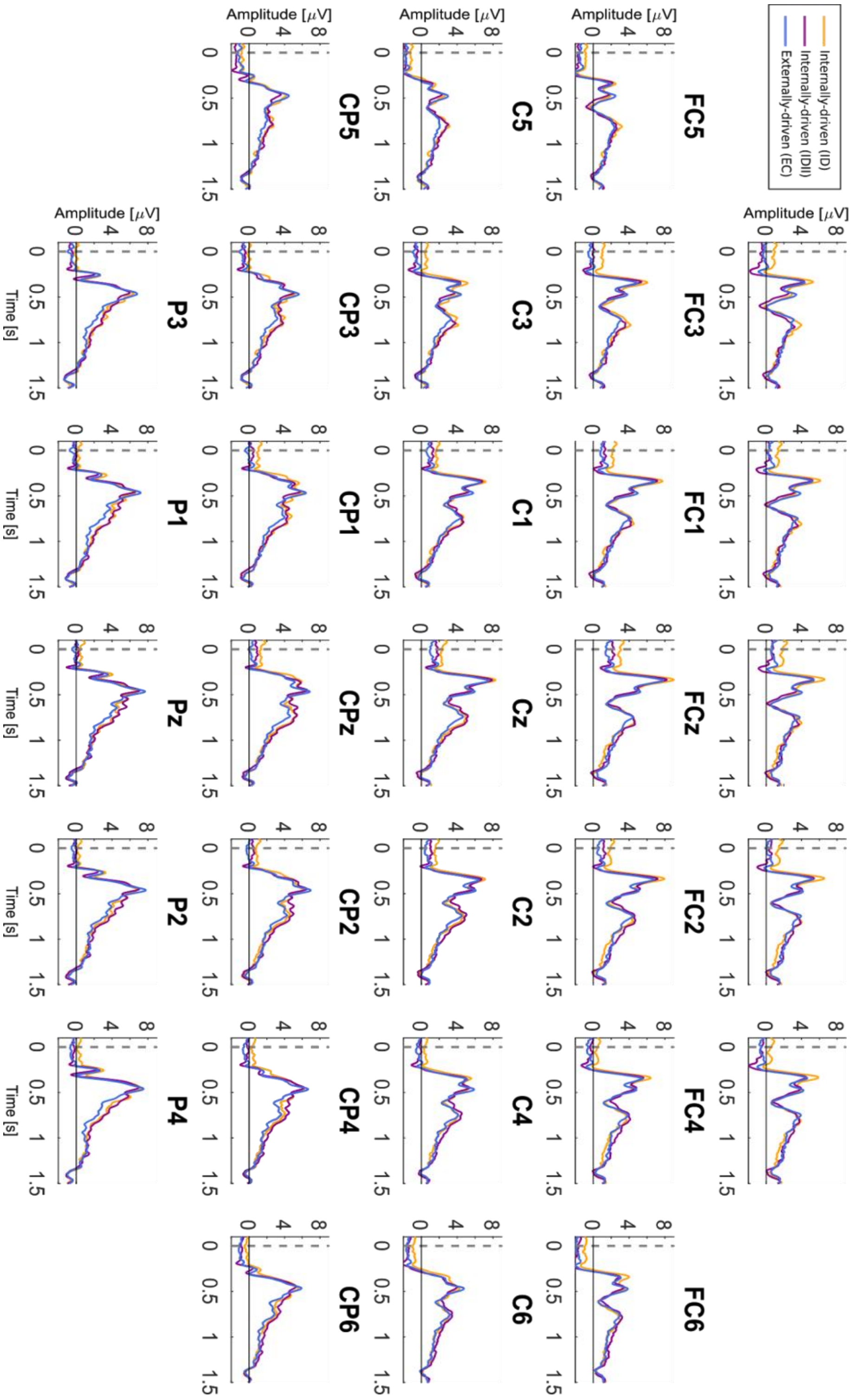

Figure I. Grand-averaged event-related potentials observed after cue presentation on an extended set of channels. In the figure, time is relative to the cue (second 0).

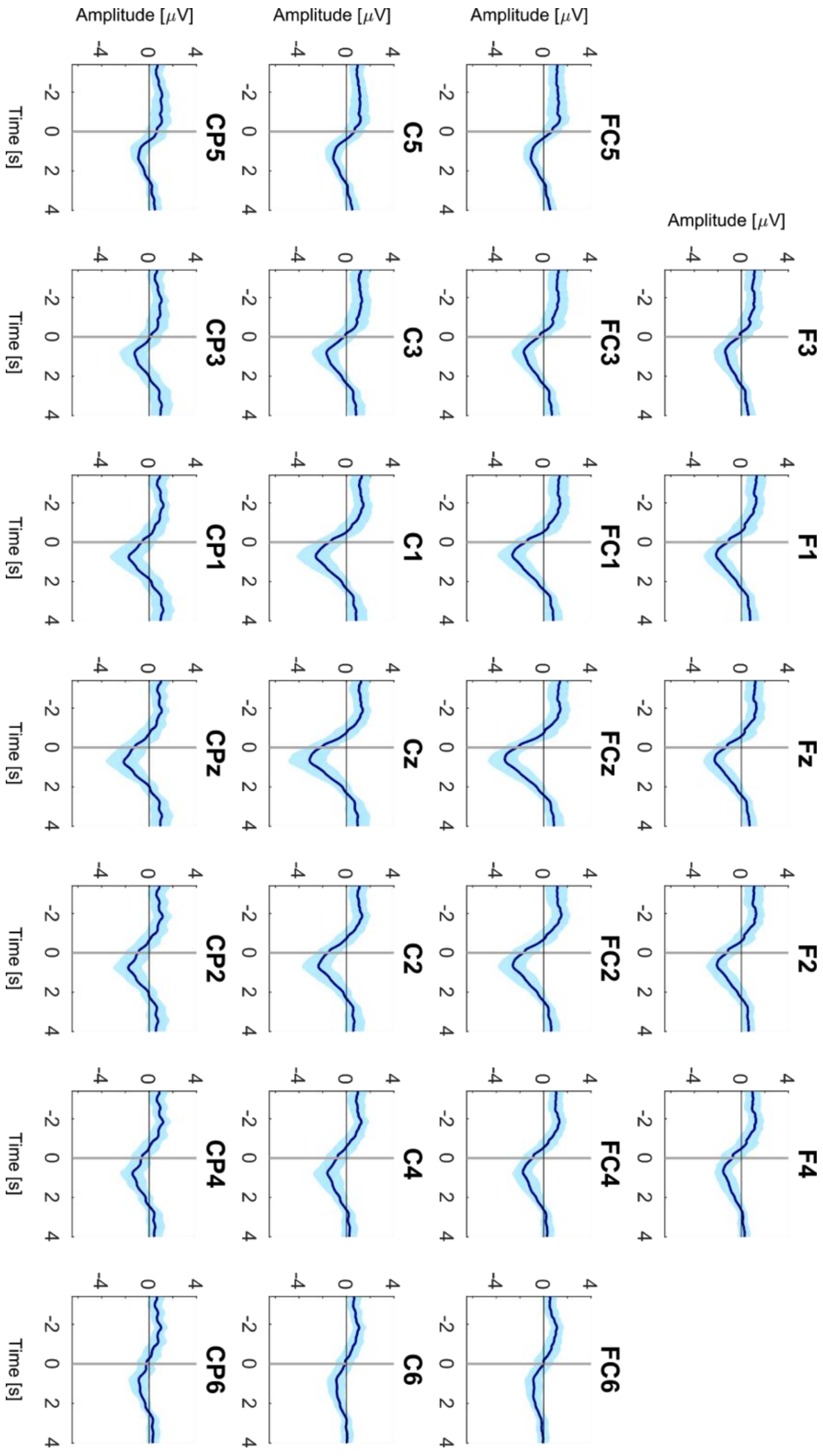

Figure II. Grand-averaged movement-related cortical potentials over the channels used for movement detection. These channels are located over somatosensory and motor areas. In the figure, time is relative to the imagination onset (IO, second 0). In light blue we show the 95% confidence interval for the mean (α = 0.05).

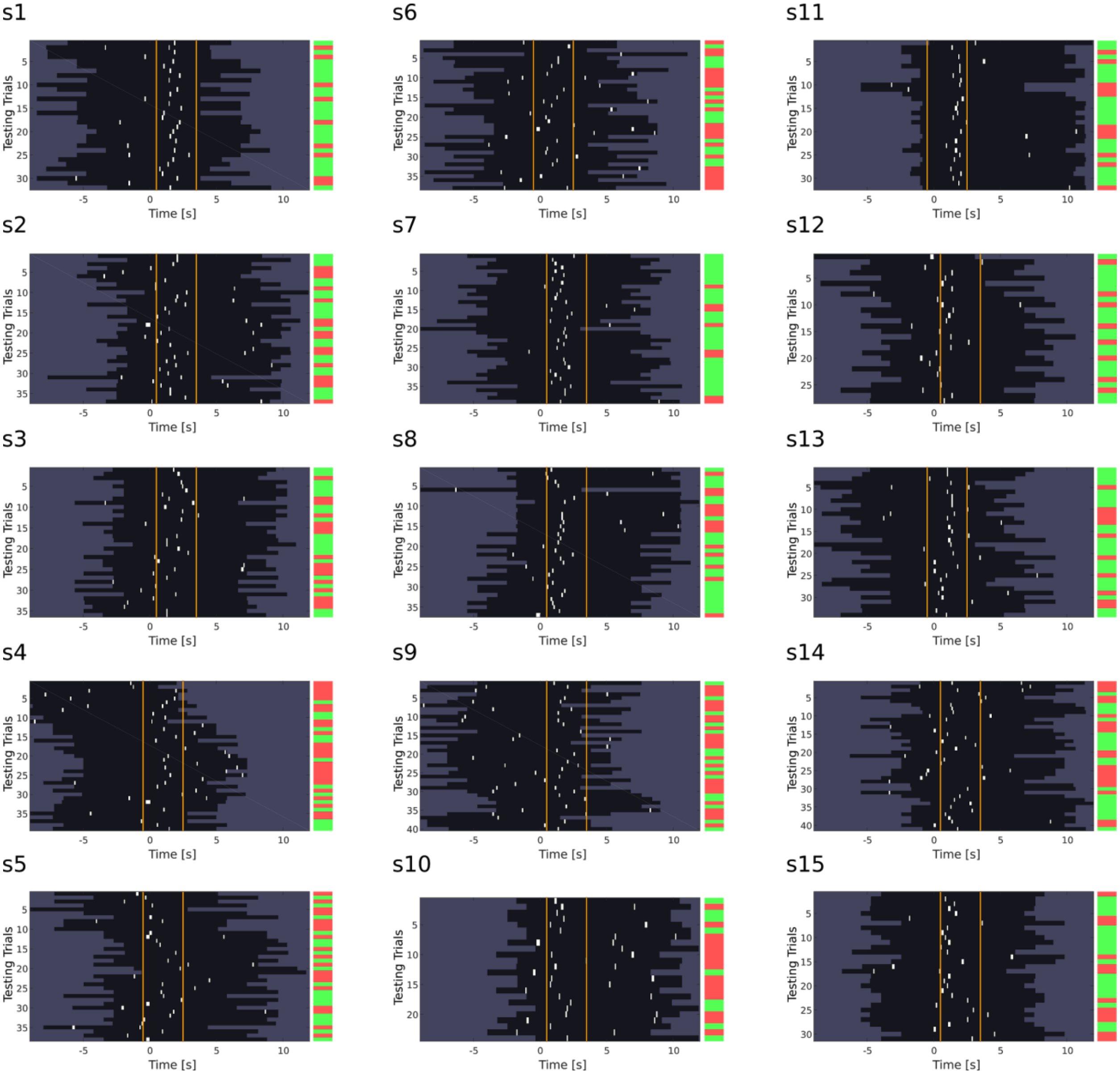

Figure III. Individual results of the asynchronous classification for one testing fold. Movement detections are marked in white over the single-trials (in black). The orange vertical lines mark the beginning and the end of the period evaluated as MI, second 0 is the imagination onset IO. The vertical bar positioned right to each of the single-trial images shows the trials marked as correct (green) and incorrect (red).

Supplementary Tables

Table I. Individual MRCP peak negativity and classification performance. In this table we show the MRCP peak negativity in channel Cz in terms of amplitude and latency (with respect to the imagination onset IO). Furthermore, the time-locked and asynchronous classification performances are shown per subject and grand-averaged. For the time-locked classification, performance was assessed in terms of accuracy. We additionally show the true positive rate (TPR), false negative rate (FNR), true negative rate (TNR) and false positive rate (FPR) in percentage. For the asynchronous classification, performance was assessed in terms of the percentage of correctly classified trials. We additionally show the positive likelihood ratio (PLR), which denotes the ratio between the true positive rate and the false positive rate.

	Subjects															
	**s1**	**s2**	**s3**	**s4**	**s5**	**s6**	**s7**	**s8**	**s9**	**s10**	**s11**	**s12**	**s13**	**s14**	**s15**	**mean ± s.d.**
MRCP peak negativity in channel Cz																
**Amplitude [μV]**	-14	-4	-3	-3	-3	-4	-4	-2	-4	*	-1	-8	-4	-3	-3	----------------
**Time [s]**	0.7	1.4	0.6	-0.4	-0.2	0.0	0.7	-0.2	0.8	*	0.6	-0.2	0.2	1.3	0.2	----------------
Time-locked classification performance																
**Accuracy [%]**	94	78	81	74	75	71	95	84	79	59	87	89	88	77	87	**81.2 ± 9.5**
**TPR [%]**	94	81	81	72	74	70	94	84	77	59	90	87	87	77	88	**81.0 ± 9.7**
**FNR [%]**	6	19	19	28	26	30	6	16	23	41	10	13	13	23	12	**19.0 ± 9.7**
**TNR [%]**	94	75	80	76	75	71	95	84	81	58	83	91	89	76	86	**80.9 ± 9.7**
**FPR [%]**	6	25	20	24	25	29	5	16	19	42	17	9	11	24	14	**19.1 ± 9.7**
Asynchronous classification performance																
**Correct trials [%]**	74	56	50	30	30	50	79	59	31	36	48	73	72	42	58	**52.5 ± 16.8**
**PLR**	32	12	9	3	3	6	35	10	3	3	8	18	16	5	9	**11.5 ± 10.1**
